# Influence of ground motion parameters on seismic response of a large-longitudinal-slope and small-radius curved girder bridge

**DOI:** 10.1371/journal.pone.0308456

**Published:** 2024-08-07

**Authors:** Shenchun Qian, Tao Cheng, Lei Mao, Xiaoxin Zhang, Zhangliang Hu

**Affiliations:** 1 Anhui Road & Bridge Engineering Co., Ltd., Hefei, Anhui; 2 Hefei Urban Construction Investment Holding Co., Ltd., Hefei, Anhui; 3 Hefei University of Technology, Hefei, Anhui; University of Zanjan, ISLAMIC REPUBLIC OF IRAN

## Abstract

The mechanical performance of curved bridges under the action of an earthquake is complex. To obtain the influence of seismic parameters on the seismic response of curved girder bridges, this paper relies on a large slope small-radius curved steel box girder bridge (LSCGB) and selects seismic wave incidence angle, vertical component of ground motion, and site category as seismic parameters to carry out nonlinear time history analysis. Based on the analysis results of the case bridge, it is shown that the torsional vibration of the first 10 modes of LSCGB is significant, the modes are dispersed, and the contribution of high-order modes of vibration cannot be ignored. The most unfavorable seismic wave incidence angle is in the direction of 45°∼60° counterclockwise Angle from the central connection line of Pier No. 1 and Pier No. 4 of the bridge. The seismic response of the curved bridge components increases with the vertical seismic intensity, and the influence on displacement responses is more significant. The basic vibration period of curved girder bridges built on soft soil sites is extended by approximately 18.23%, and the seismic response of key components increases with the softening of the site soil. Therefore, when analyzing the seismic response of LSCGBs, the influence of vertical component of ground motion and site category should not be ignored.

## 1 Introduction

### 1.1 Background

Small-radius curved girder bridges are irregular bridge structures with extremely complex forces in both plane and space. They have the characteristics of appealing aesthetics, strong crossing ability and strong adaptability, and are often found in the city viaduct and the mountain highway. The biggest feature of this kind of bridge is that due to the existence and constant change of its curvature, the flexural and torsional coupling effect is very obvious under the action of strong earthquakes, resulting in extremely complex forces in the plane and space, and it is easy to produce serious earthquake damage under the action of strong earthquakes. In the recent earthquake, the curved bridge suffered severe structural damage and even collapsed. Due to the irregularity of the structure and the rigid torsional movement of the deck, the horizontal curved bridge will cause the deck to shift and impact the expansion joint, which will suffer serious damage in the earthquake. For example, during the Wenchuan earthquake in 2008, the fifth link of Baihua Curve Bridge suffered irreparable damage such as broken pier and falling beam of the superstructure [1].

### 1.2 Literature review

Compared with the relatively mature research methods and results of straight bridges, the current research on curved bridges is still insufficient. The significant difference between curved bridges and straight bridges is the significant coupling effect between bending and torsion, as well as the different reaction forces between the inner and external bearings. Through a limited number of shaking table tests and a large number of numerical simulations to simulate complex nonlinear phenomena, more and more researchers pay attention to the seismic performance of curved bridges.

Minavand [2] conducted modal and nonlinear time-history analysis on curved box girder bridges, taking into account the effects of curvature radius, isolation bearings, and other factors on the seismic performance of curved box girder bridges. By comparing the peak displacement response and internal force response of the pier top and girder, it was found that small-radius curved box girder bridges generate greater force and displacement compared to conventional curved girder bridges. Besides, isolation bearings played a very important role in reducing force and displacement. Monzon et al. [3] conducted scaled shaking table tests on a three-span isolated curved steel I-beam bridge. Research has shown that the horizontal curvature of the girder changed the asymmetry of the structural seismic response and increased the lateral displacement at the bridge abutment. Afefy et al. [4] believed that the curvature ratio and span significantly affected the natural frequency of I-steel curved beam bridges, and the fundamental frequency significantly decreased with the increase of curvature ratio or span length. Zhang et al. [5] designed a shaking table test of a small-radius curved beam bridge with a scale ratio of 1:16 and considering the pile soil effect. The results showed that the structure had a significant torsional effect under unidirectional seismic input, and the torsional effect strengthened with the increase of PGA; The seismic response of components was more sensitive to seismic waves with relatively low frequency components; The degree of damage to the pier bottom gradually increased from short piers to high piers. The experiment also found that compared to straight bridges, the curvature radius made curved girder bridges more prone to torsional vibration and increases structural displacement. Li et al. [6] conducted scaled shaking table tests on typical curved bridges considering spatial variability of seismic motion. The test results showed that wave passage and local site effects had a significant impact on the seismic response of curved bridges. Compared to straight bridges, the curvature radius makes curved bridges more sensitive to the spatial variations of ground motions, and the damage to curved bridges may be more severe in the same earthquake. Amjadian and Agrawal [7] developed a simple and practical method to calculate the natural frequencies and vibration modes of horizontal curved bridges. At the same time, the influence of seismic impact on the rigid body motion of horizontal curved bridges under strong earthquakes was studied using parameters such as the gap between the main beam and the abutment, and the inclination angle of the main girder. Yan [8] found that local site effects amplify the seismic response of high pier small-radius curved bridges. The seismic response of high pier small-radius curved bridges was influenced by different frequency spectrum seismic waves, and there were significant differences. Based on the above results, the seismic design of high piers of small-radius curved bridges should consider the influence of multiple point excitations. Mohseni et al. [9] conducted detailed numerical simulation analysis to investigate the sensitivity of free vibration characteristics of curved high-performance steel (HPS) I-shaped steel bridges. The results showed that the fundamental frequency of natural vibration decreased with an increase in span length (L) to depth (D) ratio (slenderness = L/D), the amplitude of fundamental frequency decreased with an increase in span length to radius curvature (R) ratio (L/R), and the transverse brace increased the torsional stiffness of the girder section. Deng and Feng et al. [10,11] designed a 1/62.5 scale model of a three-span curved bridge and conducted shaking table tests to study the seismic response laws of curved girder bridges with different angles of seismic input. They also developed a result response (RRB) based calculation method, which provides theoretical and experimental verification for calculating the most unfavorable seismic input angle, and was used to evaluate the critical excitation direction of curved bridges. Mohsen [12] studied the influence of the position of the stiffness center relative to the curvature center of the bridge on the natural frequencies of different curvature curved bridges and the maximum displacement of the main beam using the OpenSEES model. It was believed that the natural frequencies usually increase with the increase of the bridge center angle, which reduces the radial displacement of the main beam but increases the tangential displacement.

For irregular Bridges, there are many factors that affect their seismic response. Wang et al. [13] analyzed the effects of seismic wave input modes, pier-girder constraint forms, and changes in width and span on the seismic response of small-radius curved beam bridges based on linear time history analysis methods. Song et al. [14] showed through research that under the condition of constant bridge deck width, the smaller the curvature radius of curved beam bridges, the greater the deck width-to-curvature radius ratio, and the greater the difference between the seismic response of curved beam bridges and the calculation results of conventional beam bridges. The difference under lateral seismic action was more significant than that under longitudinal seismic action. Uenaga et al. [15] used the seismic vulnerability method to evaluate the seismic performance of curved bridges with different curvature radii under long and short period seismic actions. The results showed that the degree of structural damage caused by long period earthquakes was higher than that caused by short period earthquakes, and when the attack angle of the seismic wave was 0°, the probability of damage to each component of the bridge was the highest. Due to the irregularity of the structure, the maximum seismic response of curved bridges is significantly related to the specified seismic input angle. In order to more conveniently determine the most unfavorable input angle, Ni et al. [16] derived a unified expression for the unidirectional seismic input unfavorable angle. In addition, Salar Farahand Tabar [17,18] found that the seismic response of cable-stayed arch bridges considering vertical seismic action is greater, especially the vertical seismic response. Compared to horizontal seismic action, the three-dimensional seismic action considering vertical seismic action increases the probability of structural damage. Li et al. [19] found through scaled shaking table tests that the seismic response of curved bridges under pulse type seismic motion is greater than that under normal seismic motion, and the impact of pulse duration on the response of curved bridges cannot be ignored.

### 1.3 Aim of the work

However, the above research only focused on horizontal curved beam bridges. On urban elevated ramps, not only are the bridge decks designed in a curved shape, but the piers also vary in height, forming a more irregular bridge structure with large longitudinal slopes and small-radius. Therefore, it is of great significance to deeply understand the damage mechanism of curved beam bridges with large longitudinal slopes and small-radius under complex seismic actions. This article takes a large longitudinal slope and small-radius curved bridge as the prototype structure. Based on numerical simulation, the dynamic characteristics and seismic response of curved bridges are analyzed, and the seismic response laws of curved beam bridges under different seismic parameters are explored. This provides a reference for the reasonable seismic design and widespread application of curved bridges.

## 2 Large-longitudinal-slope and small-radius curved girder bridge

### 2.1 Bridge description

**Fig 1:**
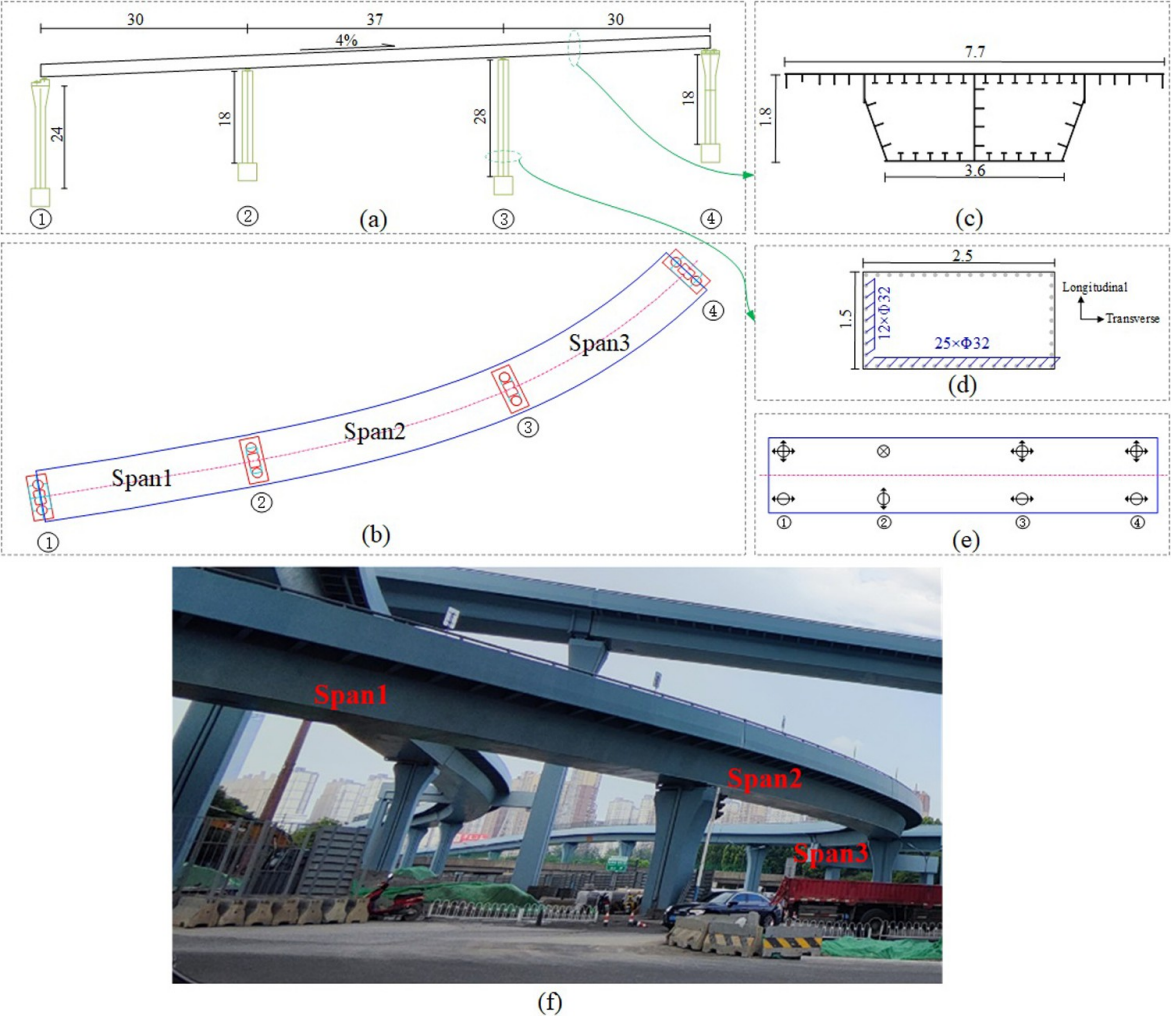
Overall layout of the case bridge (units: m). (a) Elevation. (b) Plan view. (c) Section of girder. (d) Section of pier. (e) Layout of bearings. (f) Real photos of bridge.

A municipal overhead bridge adopts the form of steel box continuous beams, and the span of the bridge near the ramp is arranged as 30m+37m+30m, as shown in Fig 1A and 1F. The deck elevation increases from pier of No. 1 to pier of No. 4 according to the gradient of 4%, which belongs to the large longitudinal slope bridge. The curvature radii of the first and third spans are 600m and 80m, respectively, and the second span is designed as a transition curve, which belongs to a small-radius curved girder bridge (Fig 1B). The standard cross-section of the girder is shown in Fig 1C, the width of the bridge deck top plate is 7.7m, and the thickness is 16-24mm; The bottom plate width is 3.6m, the beam height is 1.8m, the cantilever part is 1.6m, and the thickness is 14-24mm; T-shaped stiffeners are arranged inside the box girder, with a thickness of 12mm; The spacing between the diaphragms is 1.5m, and the material is Q345qD steel. The rectangular concrete pier poured with C40 is connected to the group pile foundation through a rectangular bearing platform, as shown in Fig 1D. The entire bridge adopts spherical steel bearings, and the arrangement of the bearings is shown in Fig 1E.

### 2.2 Finite element model (FEM)

**Fig 2:**
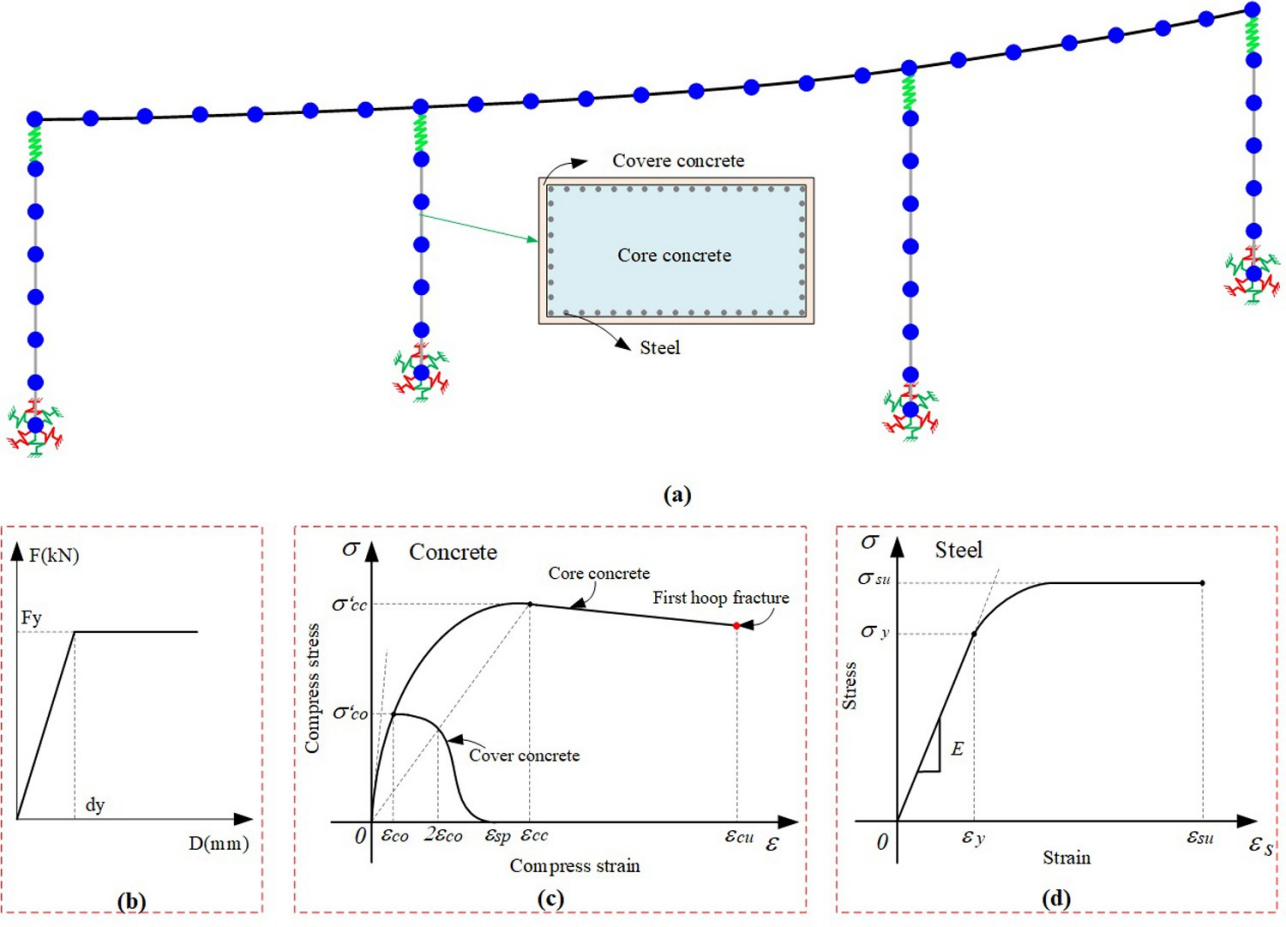
FEM. (a) FEM of case bridge. (b) Force-displacement relations of bearing. (c) Force-displacement relations of concrete. (d) Force-displacement relations of steel.

The FEM for dynamic calculation of the LSCGB is established by using OpenSEES platform [20]. The main girder is usually designed to maintain the elastic state under earthquake action. For example, shaking table tests show that the deck remains undamaged until the PGA was 1.3g [21]. So the elastic beam-column element is used to simulate the mechanical behavior of steel box girder. The bearing is a vulnerable component of the bridge under earthquake action, so the nonlinear force-displacement relationship that can consider the damage of the support is used to describe the mechanical behavior of the bearings [22,23]. Based on previous literature, the bearing adopts a double line constitutive model and is assigned a zero length element (Fig 2B), and the mechanical parameters of bearings are listed in Table 1.

**Table 1:** The mechanical parameters of bearings.

Types	Yield force F_y_ (kN)	Yield displacement d_y_ (mm)
Sliding bearings	12.26	2
Fixed bearings	122.6	2

With the increase of earthquake intensity, concrete bridge piers may experience cracking, peeling, or steel bar tensile failure. Therefore, nonlinear beam column elements are used to simulate the damage behavior of concrete bridge piers under earthquake action. The nonlinear beam column element needs to discretize the concrete pier section into protective layer concrete, core concrete, and steel fiber. The mechanical behavior of concrete was simulated using the Mander model [24] (Fig 2C), while the mechanical behavior of longitudinal steel bars was simulated using the Giuffre Mengotto Pinto uniaxial isotropic strain hardening model [25] (Fig 2D). The mechanical parameters of concrete and reinforcement of piers are listed in Tables 2 and 3. The "Matlock" method recommended in many literatures is used to simulate the interaction between pile groups and soil [26,27], that is, to calculate the stiffness of six springs composed of three translational and three rotational directions and connected to the centroid of the pile cap. This method is based on the Winkler assumption, combining the characteristics of the pile and soil, and calculating the soil spring stiffness along the length of the pile according to the principle that the lateral resistance of the pile burial depth is proportional to the deflection at that depth. The specific calculation process can be obtained in the specification [28] and will not be repeated here. The final established full bridge FEM is shown in Fig 2A.

**Table 2:** The mechanical parameters of concrete.

*σ*’_*cc*_ (MPa)	*ε*_*cc*_	*ε*_*cu*_
29.33	0.0035	0.0143

**Table 3:** The mechanical parameters of reinforcement.

*E* (GPa)	σ_*y*_ (MPa)	ε_*y*_	σ_*su*_ (MPa)	ε_*su*_
200	390	0.0019	570	0.09

### 2.3 Modal analysis

**Fig 3:**
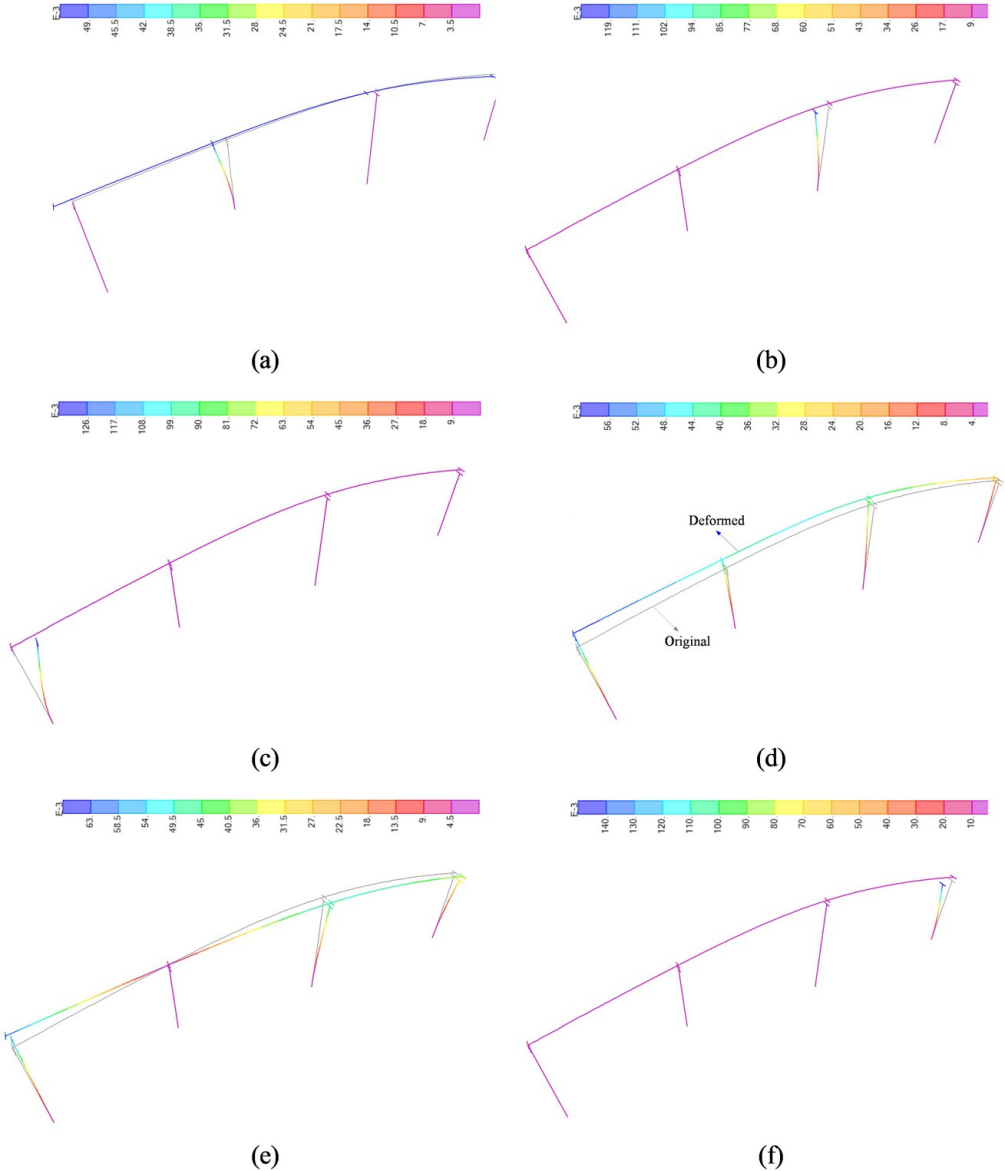
First 6 displacement modes of the structure. (a) 1st order. (b) 2nd order. (c) 3rd order. (d) 4th order. (e) 5th order. (f) 6th order.

Modal analysis is extremely important because it not only reflects the vibration characteristics of the structure, but also reveals the potential vulnerable parts of the structure. For example, Pouya Aghabeigi and Salar Farahman Tabar [29] conducted a modal analysis of a brick and stone building structure and found that the first ten torsional vibrations of the structure were more prominent. Therefore, the analysis under earthquake action showed that the structure is likely to fail due to instability. Perform modal analysis on the finite element model established in Section 2.3 to obtain its natural vibration shape and period. Due to space limitations, only the first four structural vibration modes (Fig 3) and the first ten structural vibration periods (Table 4) are listed here.

**Table 4:** Top ten modal information.

Order	Vibration period /s	Vibration mode description
1	1.141	Longitudinal vibration of the girder
2	0.837	Longitudinal vibration of the pier No. 3
3	0.662	Longitudinal vibration of the pier No. 1
4	0.537	Transverse vibration of the girder and piers
5	0.513	Transverse torsion of the girder and transverse bending vibration of the piers
6	0.374	Longitudinal vibration of the pier No. 4
7	0.335	Second order symmetric transverse bending and torsion of the girder
8	0.220	Vertical vibration of the girder
9	0.196	Second order anti symmetric transverse bending and torsion of the girder
10	0.151	Second order vertical vibration of the girder

Fig 3 depicted that the first vibration period of the bridge is 1.141s, which is longitudinal vibration. The first three orders are all longitudinal vibration, and the transverse vibration period is 0.537s until the fourth order. It is worth noting that the first ten orders have two torsional vibrations. Table 1 shows that the first ten vibration forms of the bridge are mainly longitudinal, transverse, and torsional vibrations, with vertical vibrations occurring in the eighth order and a period of 0.22 seconds. It is worth noting that pier vibration contributes to the first six modal modes. The longitudinal vibration of the pier accounts for 4 orders, and the cumulative longitudinal vibration mode mass participation coefficient reaches 0.73; the lateral vibration accounts for 2 orders, and the cumulative transverse vibration reaches 0.66.

Tables 5 and 6 list the modal parameter information with a cumulative mass participation coefficient of 0.8 for longitudinal and transverse bridge vibrations. Table 5 shows that the cumulative 6-order mass participation coefficient in longitudinal bridge vibration can only reach 0.8, while the first-order vibration mode mass participation coefficient in longitudinal bridge vibration is only 0.37. The first three orders are all longitudinal vibrations, with a cumulative mass participation coefficient of 0.64. The 6-order vibration still contributes 0.09. This means that the longitudinal vibration mode of the bridge is dispersed, and the contribution of higher-order modes to the longitudinal vibration cannot be ignored. Table 6 shows that the cumulative 5th order mass participation coefficient in the transverse bridge vibration can only reach 0.8, while the first order vibration mode mass participation coefficient in the transverse bridge direction is only 0.64, and the 16th order vibration contributes 0.06. It can be considered that the first order vibration mode in the transverse bridge direction is dominant, but the first order vibration mode mass participation coefficient is only 0.64, which also means that the contribution of higher-order modes to the transverse bridge vibration cannot be ignored. Modal analysis shows that the vibration mode of curved beam bridges with large longitudinal slopes is significantly different from that of conventional straight beam bridges, especially in the longitudinal vibration direction. Although the first three orders of curved beam bridges exhibit longitudinal drift vibration consistent with that of straight beam bridges, the mass participation coefficient of the vibration mode is relatively dispersed. This is because the longitudinal axis of a straight beam bridge is defined as the direction of the centerline connecting the two beam ends. If a curved beam bridge is still defined according to this method, the mass of the main beam of the curved beam bridge is not uniformly distributed on both sides of the longitudinal axis like a straight beam bridge, resulting in differences in the vibration modes of the two.

**Table 5:** Modal parameter of longitudinal vibration cumulative mass participation coefficient up to 0.8.

Order	Vibration period /s	Mass participation coefficient	Cumulative participation coefficient
1	1.141	0.37	0.37
2	0.837	0.14	0.51
3	0.662	0.12	0.64
6	0.374	0.09	0.73
11	0.136	0.04	0.77
14	0.108	0.04	0.81

**Table 6:** Modal parameter of transverse vibration cumulative mass participation coefficient up to 0.8.

Order	Vibration period /s	Mass participation coefficient	Cumulative participation coefficient
4	0.537	0.64	0.64
16	0.092	0.06	0.70
39	0.009	0.04	0.74
13	0.112	0.04	0.77
27	0.043	0.03	0.80

## 3 Seismic response analysis of LSCGB

### 3.1 Selection of seismic waves

The case bridge is built on hard soil, and the peak ground acceleration (PGA) in the area where the bridge site is located is 0.1g, and according to the specification [30], the average shear wave velocity of the soil layer in this site is V_s30_>500m/s. However, there is a lack of site-specific seismic hazard assessment, so 7 actual seismic waves with V_s30_>500m/s and average PGA of 0.1g are selected from the strong earthquake record database of the PEER [31]. It should be noted that this section aims to obtain the seismic response law of LSCGBs rather than the seismic response values of specific components, which is less affected by the selection of seismic waves.

**Fig 4:**
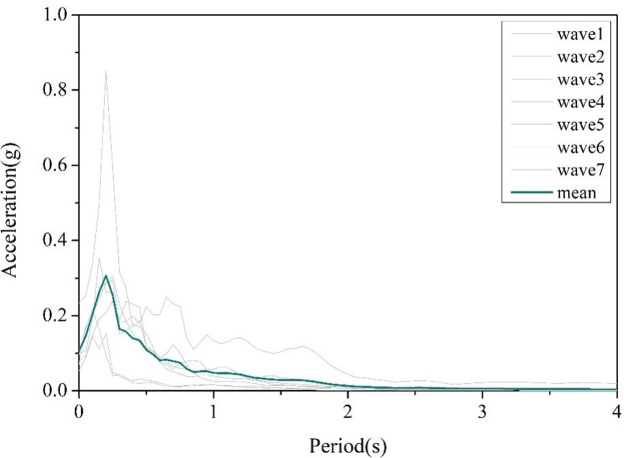
Response spectra and average response spectra of 7 selected seismic waves.

The seismic wave information is shown in Table 7, and the response spectra and average response spectra of 7 seismic waves are depicted in Fig 4.

**Table 7:** Detailed information of 7 seismic waves on a hard site.

No.	Earthquake	Year	Station	Magnitude	Distance(km)	PGA(g)
wave1	N. Palm Springs	1986	ARM270	6.06	38.22	0.097
wave2	N. Palm Springs	1986	AZF225	6.06	42.17	0.115
wave3	N. Palm Springs	1986	H01090	6.06	54.67	0.054
wave4	N. Palm Springs	1986	H02090	6.06	48.92	0.071
wave5	Northridge-01	1984	ALH090	6.69	35.66	0.101
wave6	Northridge-01	1994	BAL090	6.69	71.30	0.080
wave7	Northridge-01	1994	MTW000	6.69	35.53	0.234

### 3.2 Excitation of seismic waves

Usually, the seismic waves of a straight bridge are input in the defined longitudinal and transverse directions, indicating that the definition of the longitudinal and transverse directions of the bridge is directly related to the seismic input. Due to the difference between curved bridges and straight bridges, the most unfavorable input of longitudinal and transverse bridge directions under unidirectional seismic action needs to be defined first.

**Fig 5:**
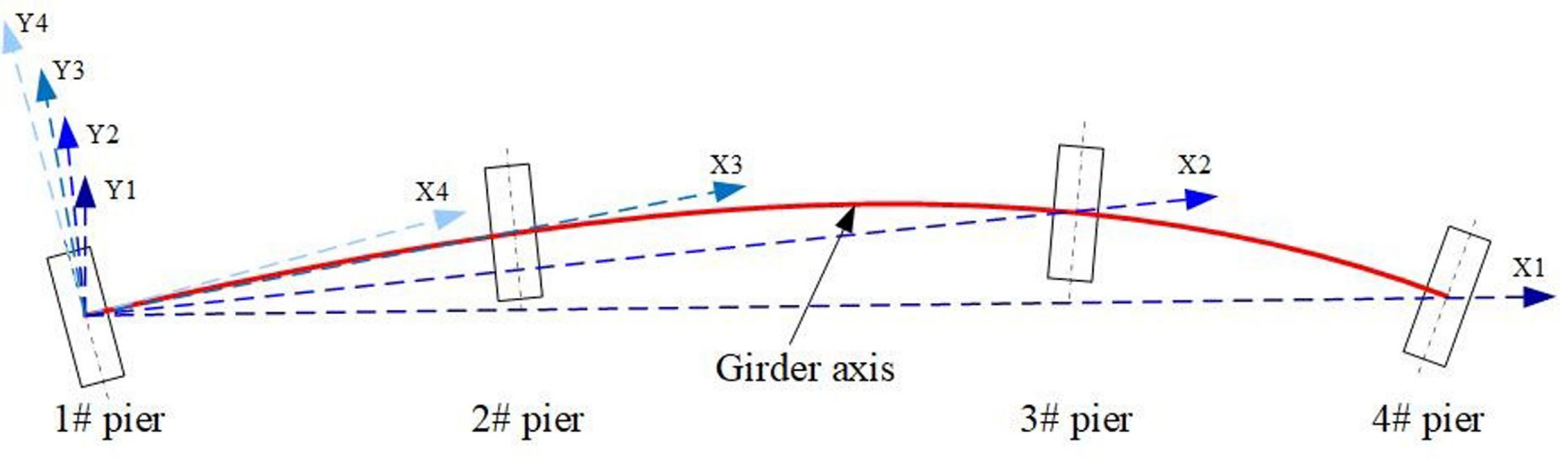
Schematic diagram of longitudinal and transverse axes of curved girder bridge.

According to the method of defining the longitudinal direction of a straight bridge, the direction of the central connecting line of Pier 1 and Pier 4 in the case of a curved bridge is defined as the longitudinal axis X1, the direction of the central connecting line of Pier 1 and Pier 3 is defined as the longitudinal axis X2, the direction of the central connecting line of Pier 1 and Pier 2 is defined as the longitudinal axis X3, and the tangent direction between the centerline of Pier 1 and the girder axis is defined as the longitudinal axis X4; The perpendicular direction of each longitudinal axis is defined as the transverse direction, presented in Fig 5.

### 3.3 Seismic response analysis

Under the action of earthquakes, curved bridges may experience longitudinal and transverse displacement of the main beam, as well as damage to the bridge piers [5]. Therefore, bearing displacement (relative displacement of piers and girder), pier top displacement and pier bottom bending moment are selected as the key engineering demand parameters (EDPs). Seven seismic waves are loaded in the longitudinal and transverse axes of the four types of bridge defined in Fig 5, and the average of the seven seismic waves is taken as the final EDPs.

**Fig 6:**
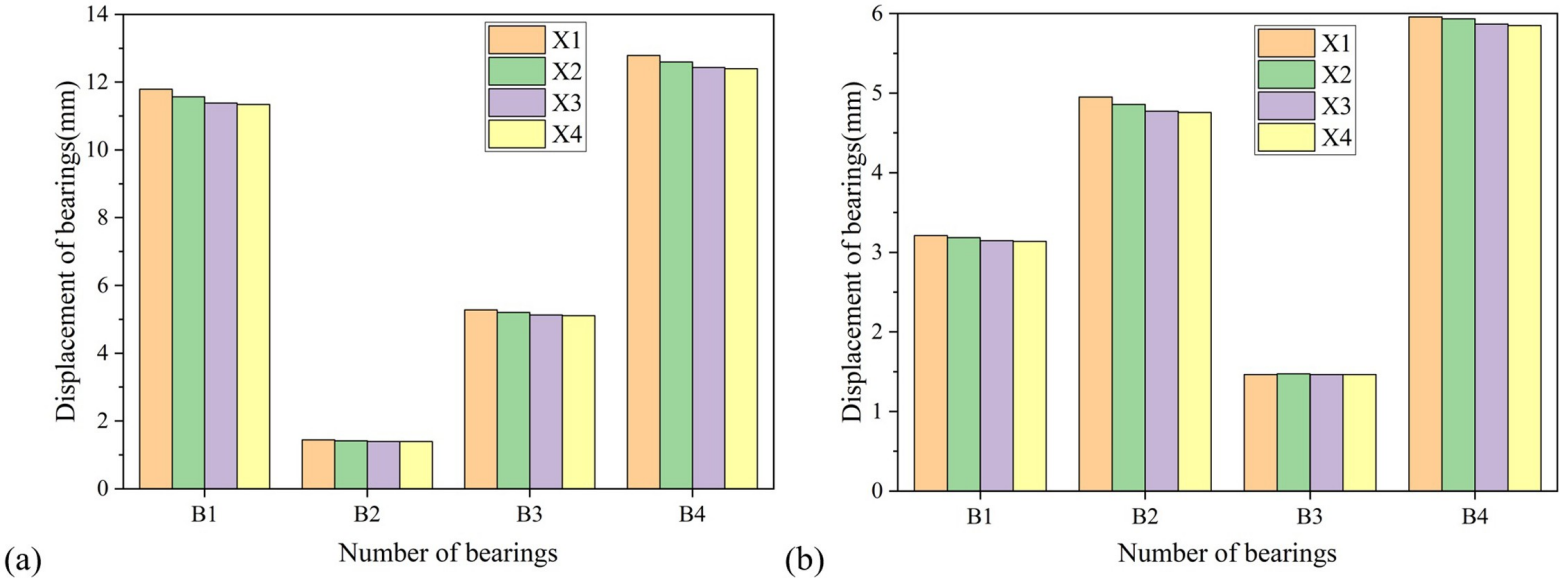
Variation of bearing displacement with the longitudinal axis of the bridge. (a) Longitudinal direction. (b) Transverse direction.

**Fig 7:**
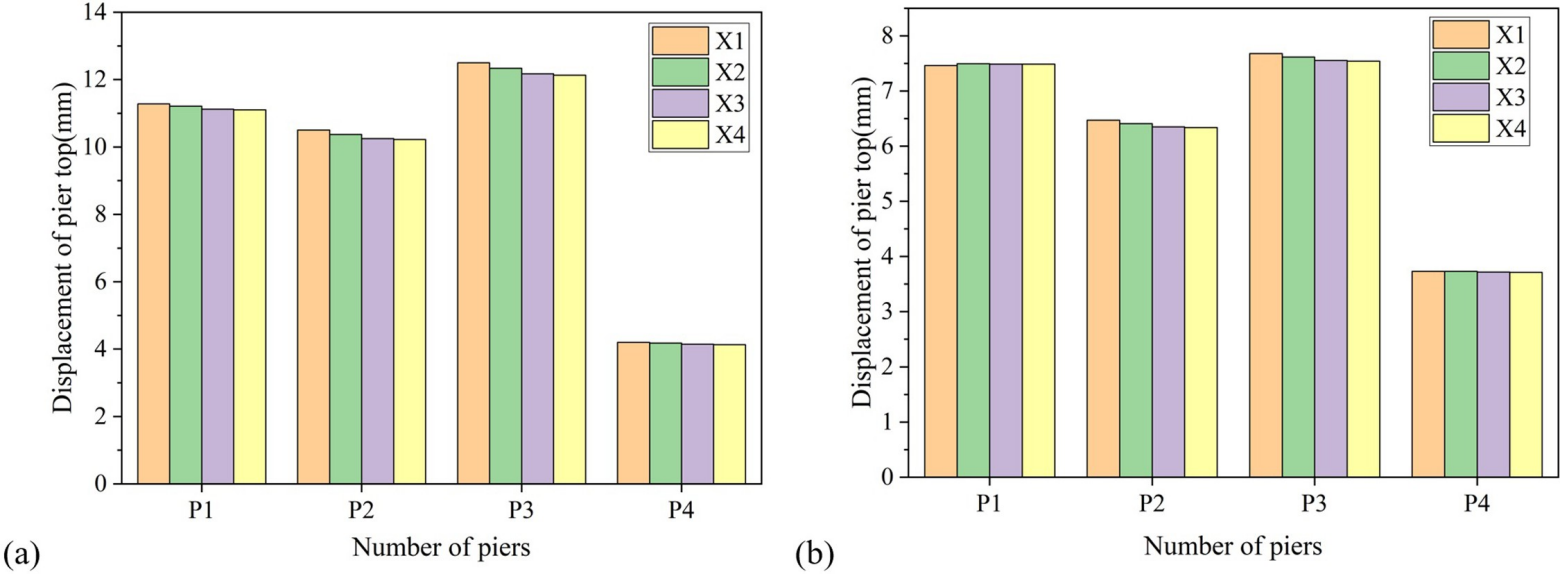
Variation of pier top displacement with the longitudinal axis of the bridge. (a) Longitudinal direction. (b) Transverse direction.

**Fig 8:**
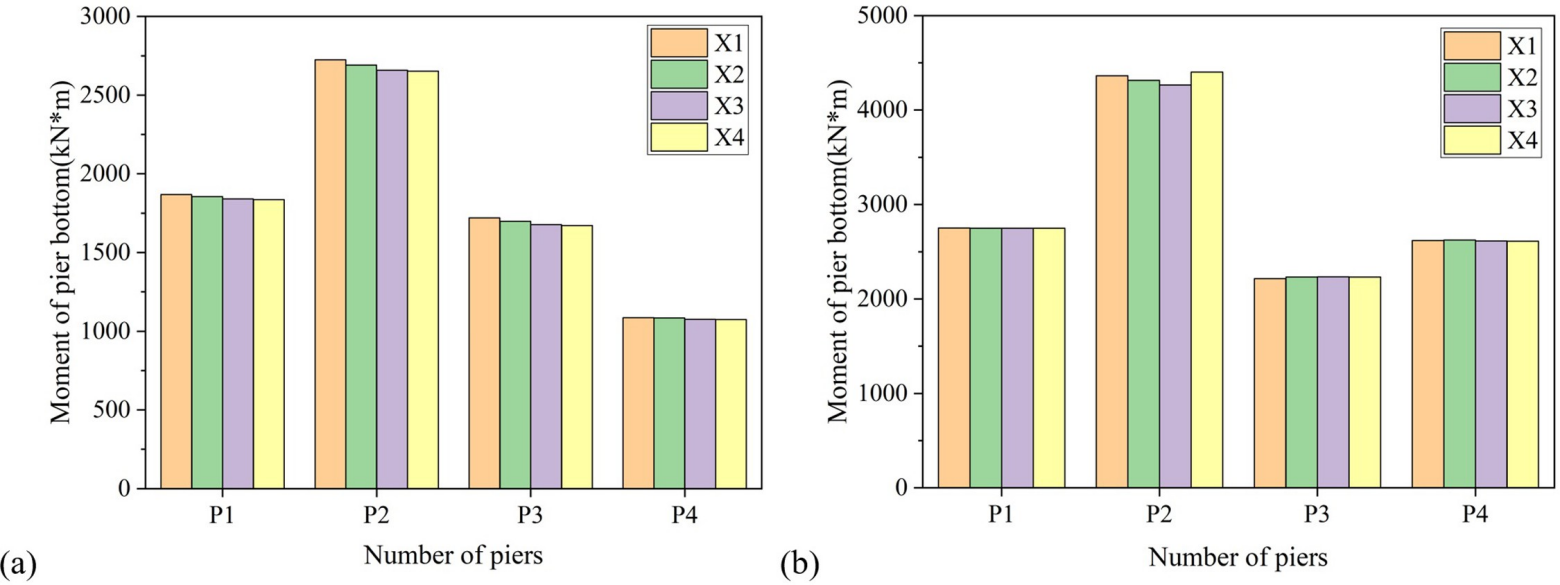
Variation of pier bottom bending moment with the longitudinal axis of the bridge. (a) Longitudinal direction. (b) Transverse direction.

Figs 6–8 show the trend of bearing displacement, pier top displacement and pier bottom bending moment with respect to the longitudinal axis of the bridge. From the figures, it can be seen that the seismic response of each component shows a decreasing trend with the increase of the defined longitudinal axis number of the bridge, that is, the longitudinal bridge response obtained by loading seismic waves along the X1 axis and the transverse bridge response obtained by loading perpendicular to the X1 axis are both the largest. This means that the axis formed by the connecting line between the center of pier No. 1 and pier No. 4 of the cased curved girder bridge is the most unfavorable longitudinal input direction.

Further analysis of Figs 6–8 shows that the longitudinal displacement of movable bearings is greater than that of fixed bearings, while the transverse bridge displacement of fixed bearings is only less than that of movable bearings No. 4. Overall, the displacement of bearings No. 4 is the largest. Taking the X1 axis as an example, when seismic waves are loaded along the X1 axis, their longitudinal displacement reaches 12.80mm, and when loaded perpendicular to the X1 axis, their transverse displacement reaches 5.96mm.

Fig 7 shows that the displacement of the top of the pier increases with the increase of the height of the pier, that is, the displacement of the top of the pier No. 3 is the largest, followed by that of the pier No. 1, and the displacement of the top of the pier No. 2 and No. 4 is the smallest. The displacement at the top of the pier is an absolute physical quantity, which changes with the height of the pier, making it difficult to compare the damage situation of individual bridge piers. Therefore, the displacement at the top of the pier is divided by the height of the pier, and the resulting drift ratio at the top of the pier is shown in Table 8. It can still be seen that the pier top drift ratio of all piers obtained by taking X1 as the longitudinal axis is the largest, and the pier top drift ratio of pier No. 2 is the largest among all piers, indicating that pier No. 2 is the first to fail compared with other piers. This is because the No. 2 pier is arranged with fixed supports, which bear more horizontal inertia force to the pier under the action of earthquakes, resulting in its pier top drift ratio is significantly greater than other piers.

**Table 8:** Drift ratio of the pier top.

Pier	Longitudinal				Transverse			
	X1	X2	X3	X4	X1	X2	X3	X4
P1	0.047%	0.047%	0.046%	0.046%	0.031%	0.031%	0.031%	0.031%
P2	0.058%	0.058%	0.057%	0.057%	0.036%	0.036%	0.035%	0.035%
P3	0.045%	0.044%	0.043%	0.043%	0.027%	0.027%	0.027%	0.027%
P4	0.023%	0.023%	0.023%	0.023%	0.021%	0.021%	0.021%	0.021%

Note: Drift ratio = (the displacement of the top of the piers)/ (the height of the piers).

The bottom bending moment diagram of pier shown in Fig 8 more clearly reflects that the bottom bending moment of pier No. 2 is the largest, the bottom bending moment of pier No. 1 is the largest among all movable piers, and the distribution law of bending moment borne by each pier is the same as the top drift ratio of pier.

## 4 Influence analysis of key parameters of ground motions

From the analysis in the previous section, it can be seen that not only does the vibration characteristics of LSCGB differ from straight girder bridges, but the response law under unidirectional seismic input is complex. The above literature review shows that for irregular bridges, the seismic response is greatly affected by the input angle of ground motions, vertical ground motion components and site type (site characteristic period) [15–18,32,33]. Therefore, it is urgent to study the impact of more complex seismic parameters on their seismic responses. In view of this, this section takes the ground motion incident angle, vertical seismic intensity and site category as key seismic parameters to conduct sensitivity analysis of the seismic response of key components of LSCGB, and explore the influence of the above parameters on the seismic response of such bridges.

### 4.1 Influence of ground motion incident angle

**Fig 9:**
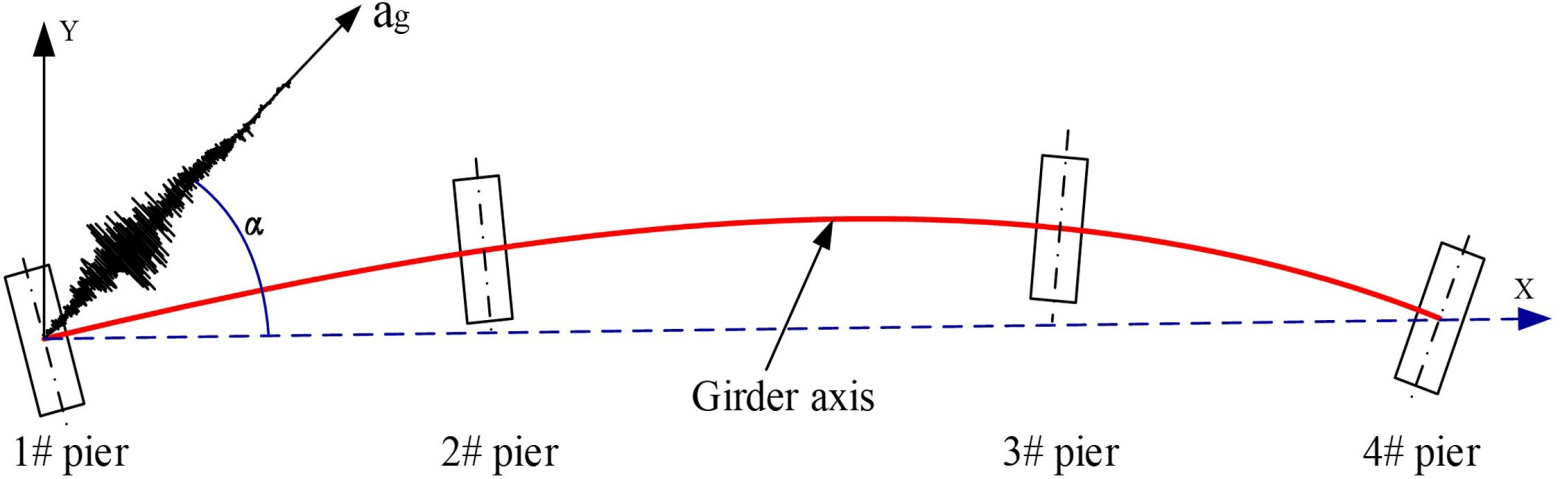
Incident angle of ground motions.

Select X1 as the longitudinal axis of the bridge (the reference axis for seismic wave loading, with an incident angle of 0°), and increase the incident angle counterclockwise in increments of 15° within the range of 0° to 90°, as shown in Fig 9. Observe the changes in bearing displacement, pier top displacement, and pier bottom bending moment with respect to the angle of attack of the seismic waves, based on the seismic input of 7 seismic waves in Section 3.1.

**Fig 10:**
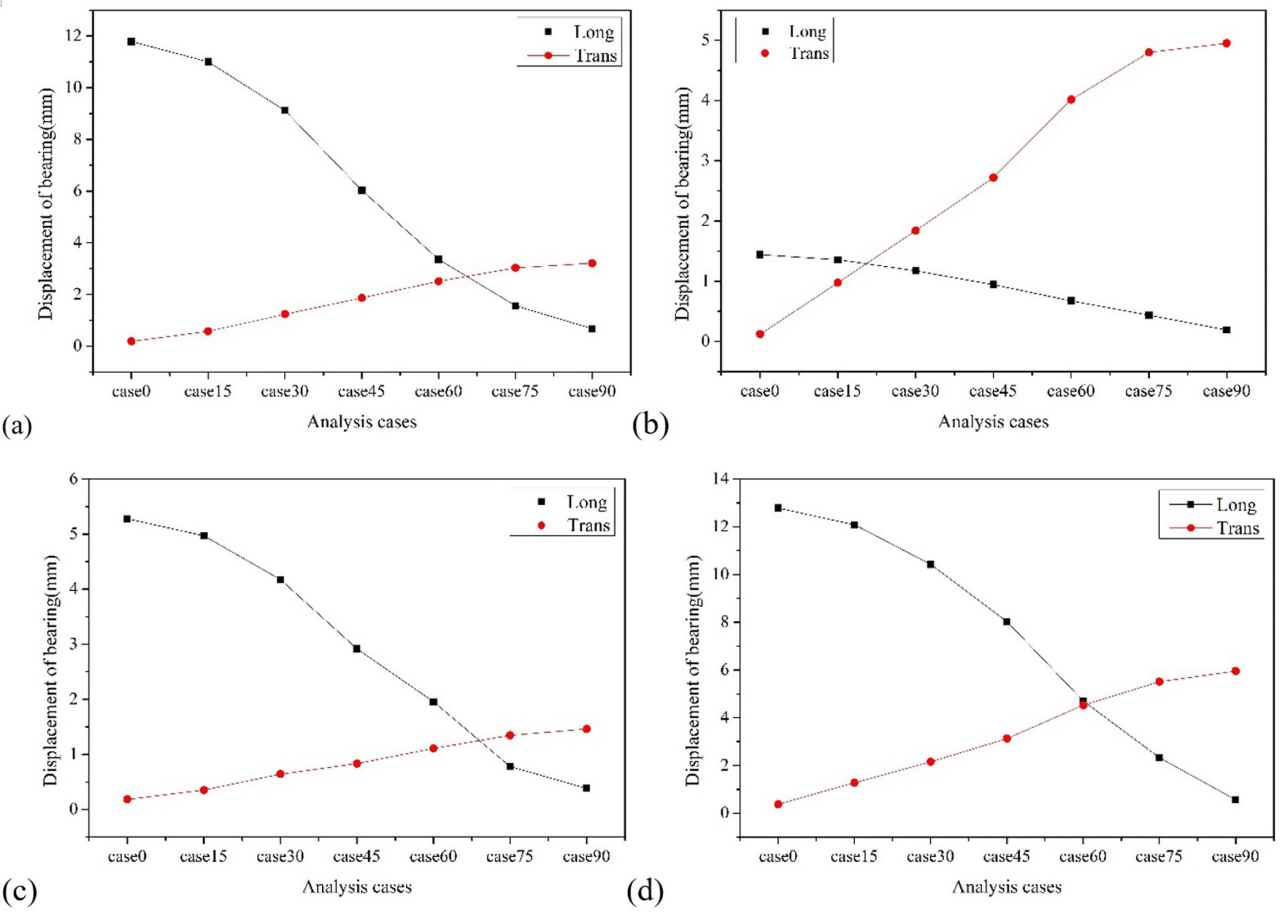
Diagram of the variation of bearing displacement with incident angle. (a) Bearing No. 1. (b) Bearing No.2. (c) Bearing No.3. (d) Bearing No.4.

Fig 10 shows the variation of the input angle of seismic wave of the bearing displacement. The displacement of all bearings follows a consistent pattern with the angle of attack of seismic waves, that is, the longitudinal displacement of the bearings decreases with the increase of the incident angle, while the transverse displacement of the bearings increases with the increase of the incident angle. Taking bearing No. 4 as an example, when the angle between the loading seismic wave and the longitudinal axis (X1) of the bridge is 0°, the longitudinal displacement reaches 12.80mm, and the transverse displacement is only 0.38mm. When the angle with the longitudinal axis is 90°, the transverse displacement reaches 5.96mm, while the longitudinal bearing displacement is only 0.56mm.

In addition, between an angle of 60° to 75°, there is a cross phenomenon in the longitudinal and transverse displacement of the bearings. Taking the bearing No. 4 as an example, when the angle between the loaded seismic wave and the X1 is 60°, the longitudinal and transverse displacement of the bearing are 4.70mm and 4.53mm, respectively. This indicates that the seismic wave incidence at this angle causes the longitudinal and transverse displacement of the bearing to be equal. It is worth noting that after the local seismic wave loading exceeds 75°, the increase in transverse displacement of the bearing tends to be gentle, but the longitudinal displacement of the bearing still drops sharply. Therefore, only from the perspective of longitudinal and transverse displacement of the bearings, the angle between the seismic wave and the longitudinal axis at 60° is the most unfavorable incident angle for the case bridge.

**Fig 11:**
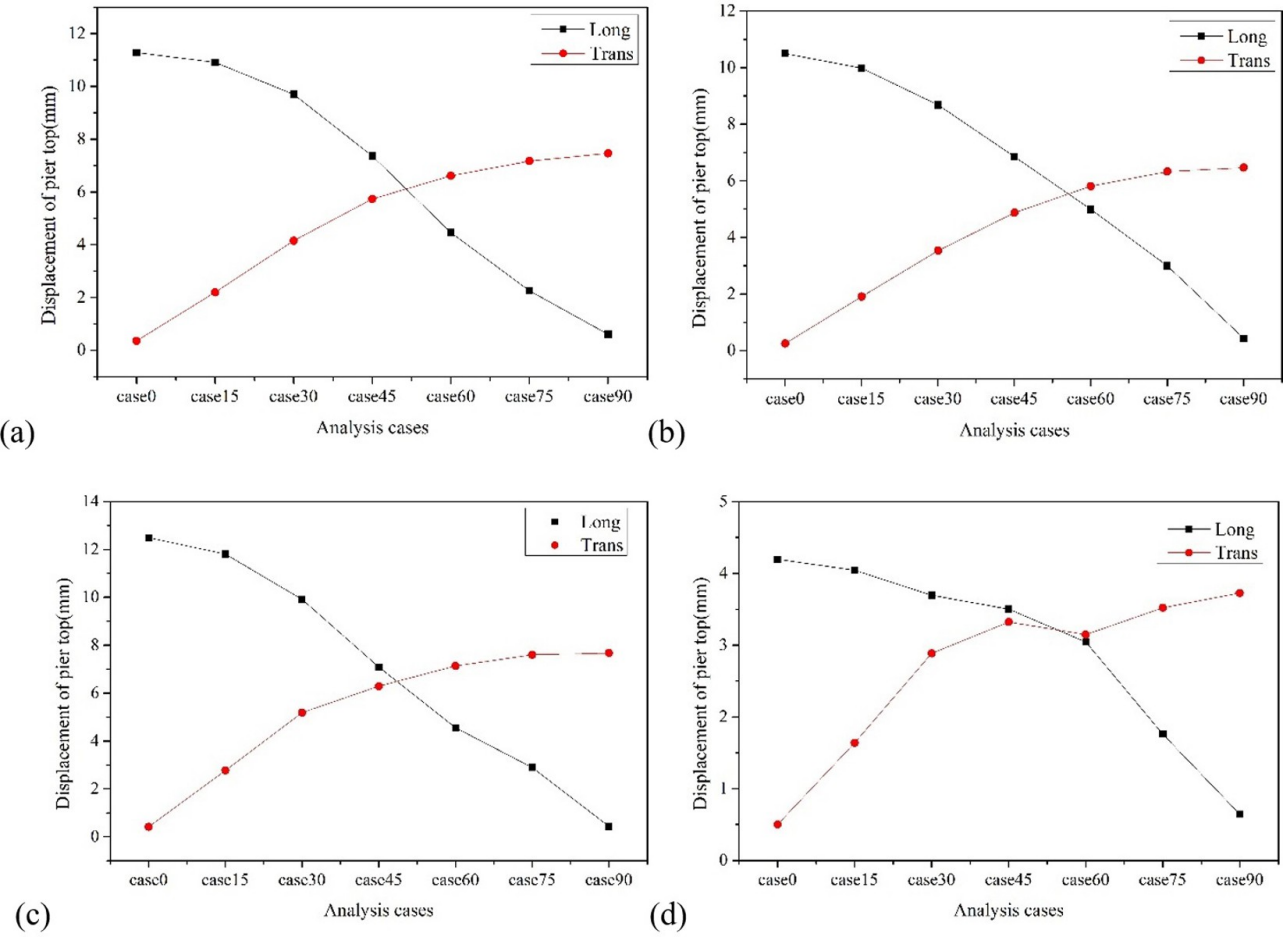
Diagram of the variation of pier top displacement with incident angle. (a) Pier No. 1. (b) Pier No. 2. (c) Pier No. 3. (d) Pier No. 4.

Fig 11 shows the variation of the input angle of seismic wave of the pier top displacement. The displacement at the top of all piers follows a consistent pattern with the direction of seismic wave loading, that is, the longitudinal displacement at the top of the pier decreases with the increase of the incident angle, and the transverse displacement at the top of the pier increases with the increase of the incident angle. Taking pier No. 3, which has the largest displacement at the top of the pier, as an example, the maximum longitudinal displacement at the top of the pier occurs at a seismic wave attack angle of 0°, which is 12.50mm; The maximum longitudinal and transverse bending moment occurs at a seismic wave attack angle of 90°, which is 7.68mm. It is worth noting that when the X1 axis is used as the longitudinal axis of the bridge, there is a cross phenomenon between the longitudinal and transverse displacement of the pier towards the pier top at the attack angle of 45° to 60°. Taking pier No. 3 as an example, when the local seismic wave input angle is 49°, the longitudinal and transverse displacement of the pier top is equal, about 6.51mm, which means that at this seismic wave attack angle, the longitudinal and transverse displacement of the pier top excited is relatively large.

**Fig 12:**
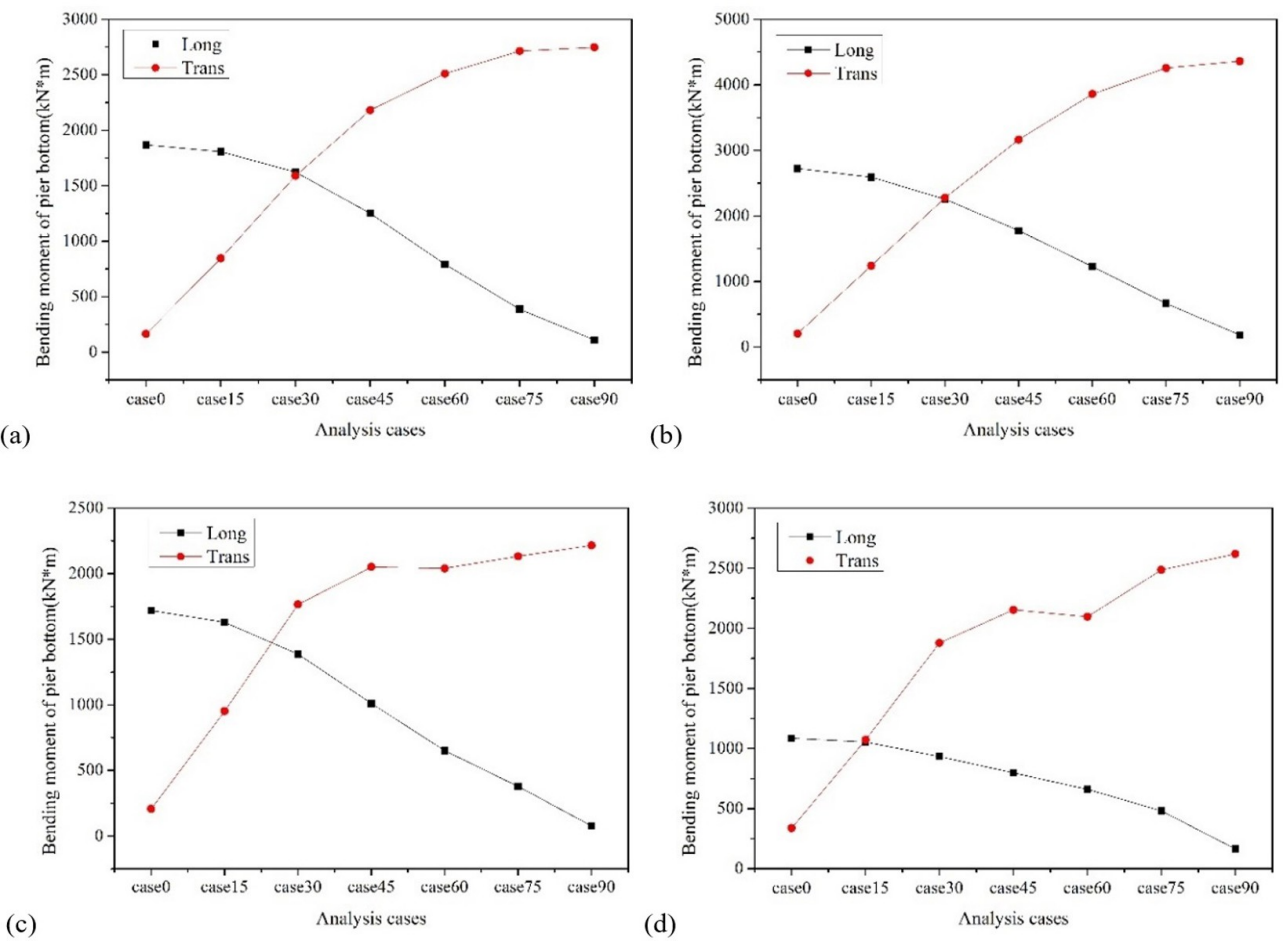
Diagram of the variation of pier bottom bending moment with incident angle. (a) Pier No. 1. (b) Pier No. 2. (c) Pier No. 3. (d) Pier No. 4.

Fig 12 shows the variation of the input angle of seismic wave of the pier bottom bending moment. It can be observed that the bending moment at the bottom of all piers follows a consistent pattern with the direction of seismic wave loading, that is, the longitudinal bending moment of piers decreases with the increase of the incident angle, and the transverse bending moment of piers increases with the increase of the incident angle. Taking pier No. 2 as an example, the maximum longitudinal bending moment of the pier occurs at a seismic wave attack angle of 0°, which is 2723Kn·m; The maximum longitudinal and transverse bending moment occurs at a seismic wave attack angle of 90°, which is 4362 Kn·m.

It ©s worth noting that when the X1 axis is used as the longitudinal axis, there is a cross phenomenon between the longitudinal and transverse bending moments of the pier at the incident angle of 15° to 30°. Taking pier No. 2 as an example, when the local seismic input angle is 30°, the longitudinal and transverse bending moments of the pier are 2255 and 2278 KN·m, respectively. Although the transverse bending moment of pier increases with the increase of the incident angle, the amplitude of the increase decreases with the increase of the input angle. When the incident angle of local seismic wave is about 60°, the sum of the longitudinal and transverse bending moments of pier reaches the maximum value, that is, 5087 KN·m, which means that this angle is the most unfavorable incident angle from the perspective of pier’s responses.

According to the criterion that the longitudinal and transverse responses of the bearing displacement, pier top displacement and pier bottom bending moment are all maximized, the most unfavorable horizontal seismic wave incident angle range for the LSCGB is 45° to 60° counterclockwise from the longitudinal axis of the bridge.

### 4.2 Influence of vertical seismic intensity

For curved girder bridges with large longitudinal slopes and small-radius, the uneven distribution of pier height and superstructure mass often results in significant differences in the weight borne by the piers on the superstructure. Vertical earthquakes exacerbate the uncertainty of this mass distribution, ultimately leading to uncertainty in the response of the bearings and piers [22]. Therefore, this section will analyze the impact of vertical seismic intensity on the seismic response of key components of LSCGB by increasing the ratio of vertical to horizontal seismic intensity.

**Fig 13:**
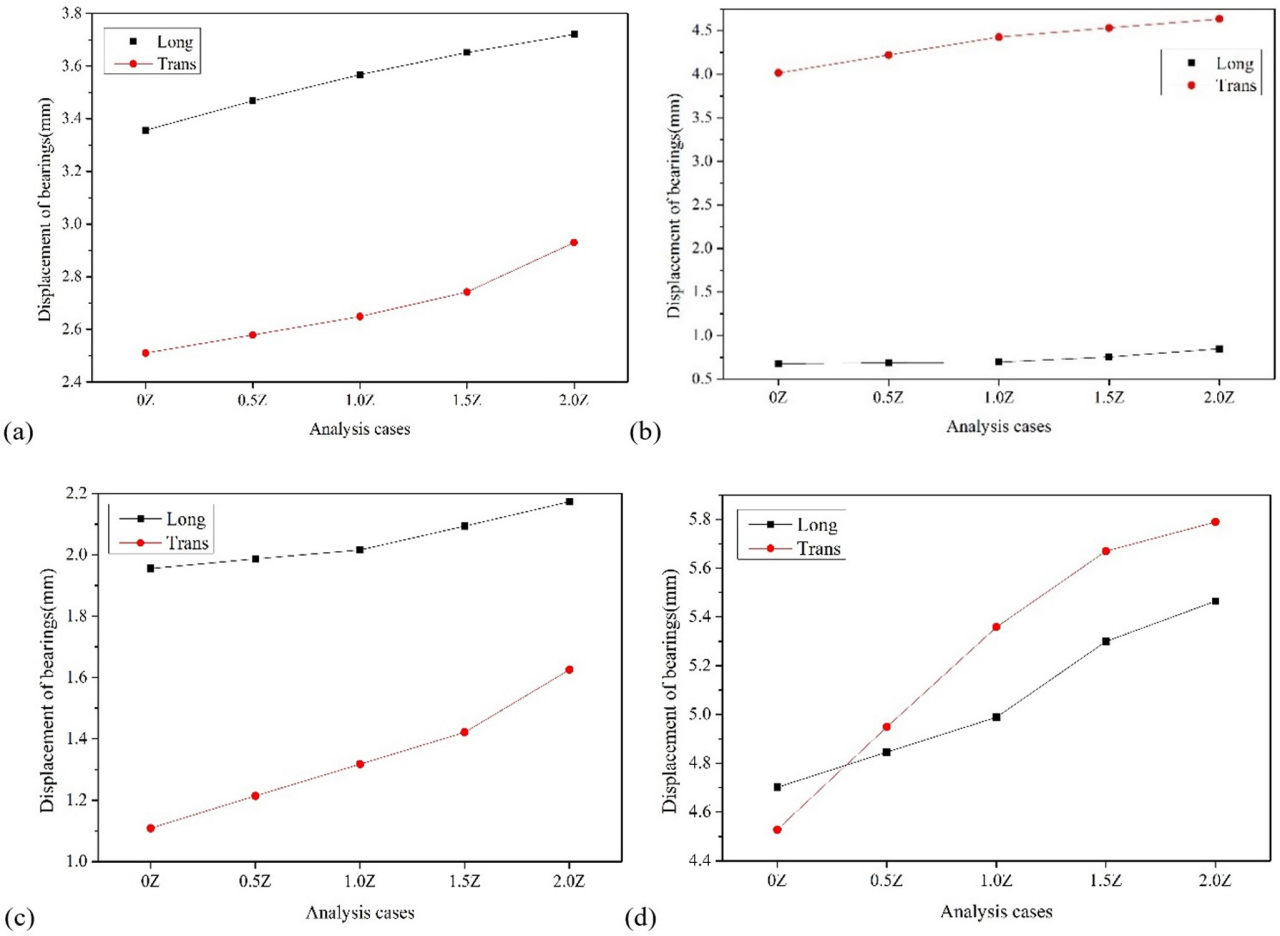
Diagram of the variation of bearing displacement with vertical seismic intensity. (a) Bearing No. 1. (b) Bearing No.2. (c) Bearing No.3. (d) Bearing No.4.

**Fig 14:**
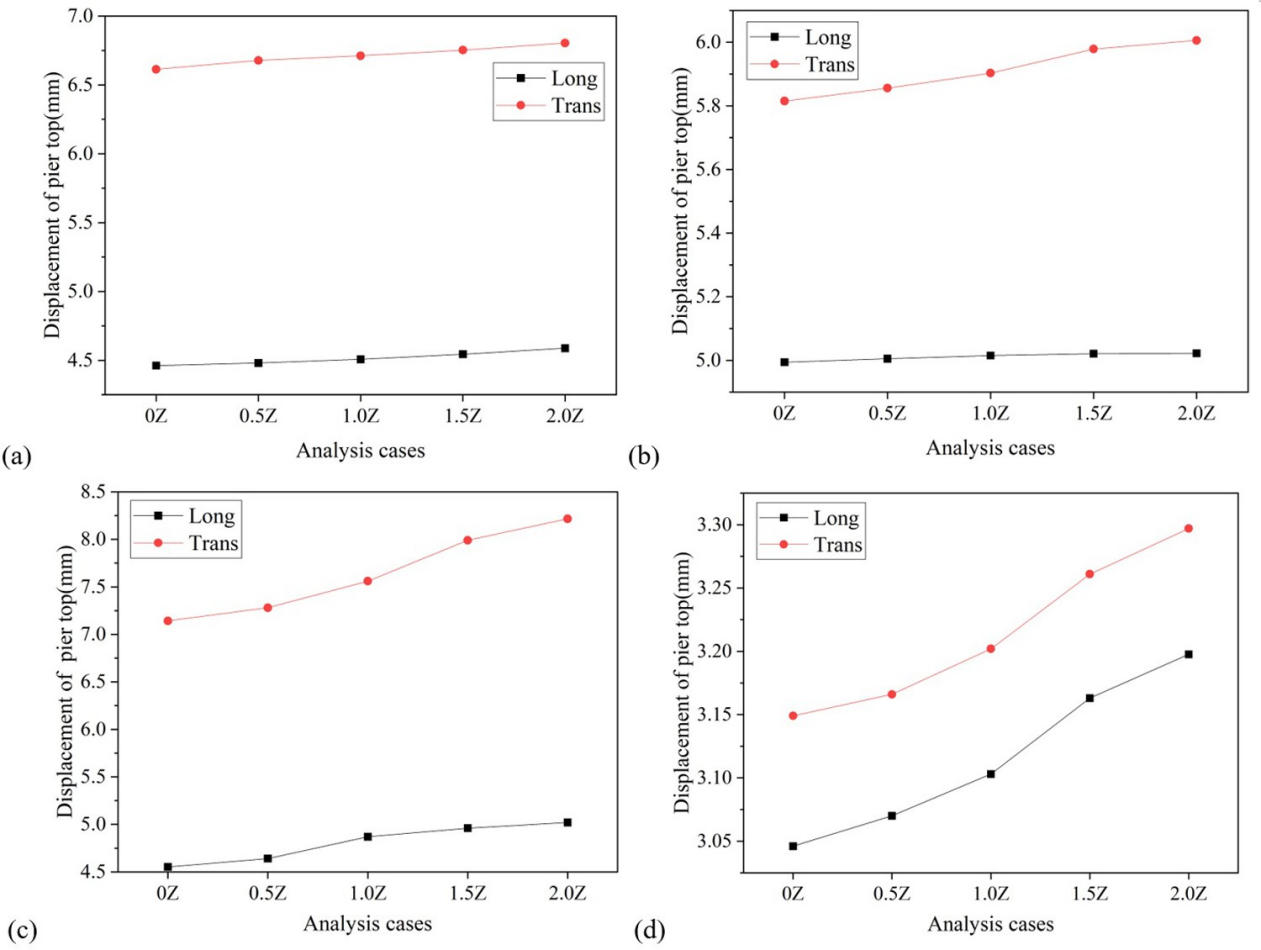
Diagram of the variation of pier top displacement with vertical seismic intensity. (a) Pier No. 1. (b) Pier No. 2. (c) Pier No. 3. (d) Pier No. 4.

**Fig 15:**
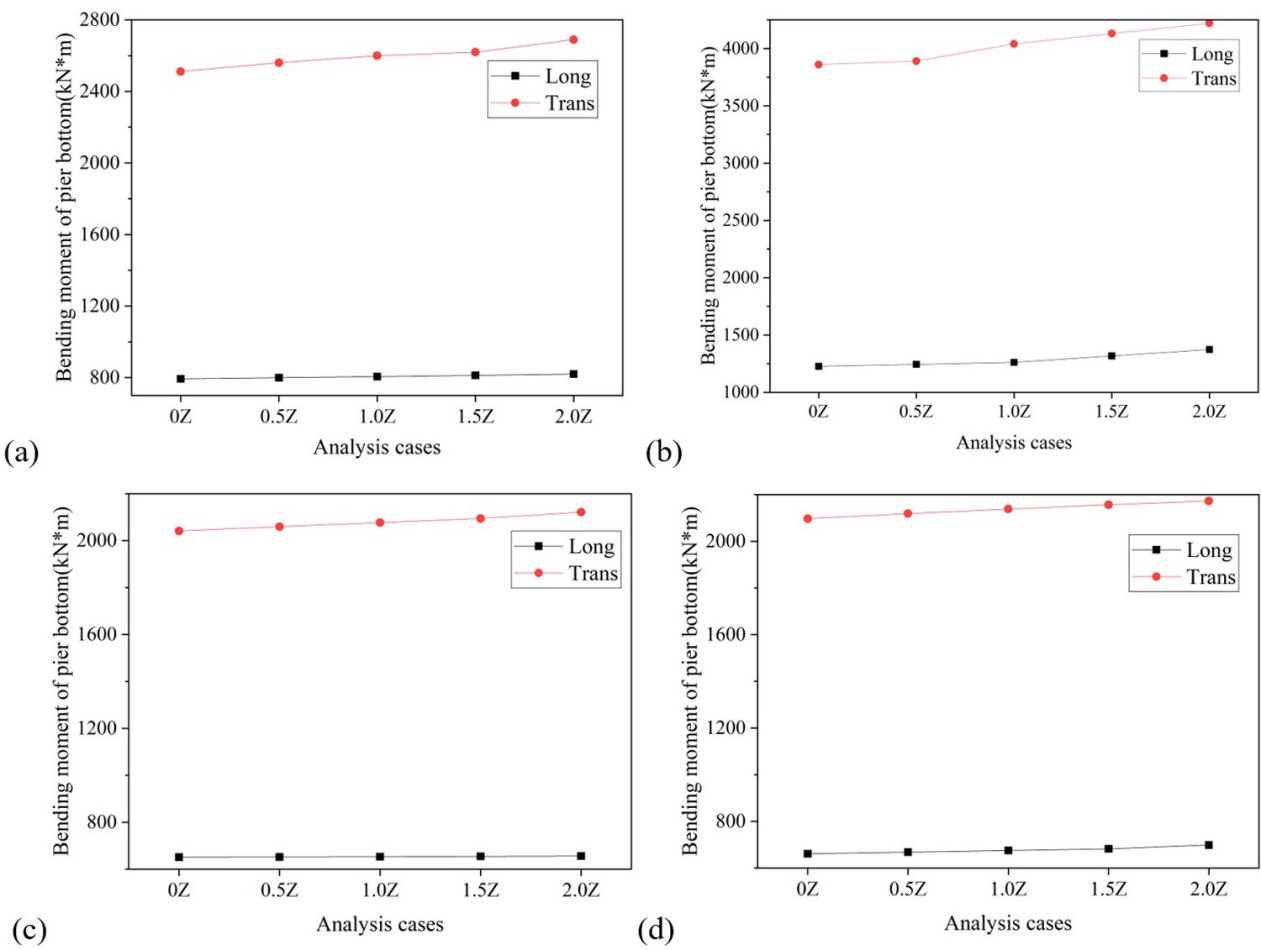
Diagram of the variation of pier bottom bending moment with vertical seismic intensity. (a) Pier No. 1. (b) Pier No. 2. (c) Pier No. 3. (d) Pier No. 4.

Figs 13–15 show the variation trend of bearing displacement, pier top displacement, and pier bottom bending moment with vertical seismic intensity. Overall, the seismic response of components increases with the increase of vertical seismic intensity. Taking bearing No.4 as an example, the longitudinal and transverse bearing displacements induced by vertical ground motion with 2 times the horizontal seismic intensity are 5.47mm and 5.79mm, respectively, which increase by 16.23% and 27.87% compared with the displacement without vertical ground motion. Taking the top displacement of pier No. 3 as an example, the vertical and transverse displacement of pier top induced by vertical ground motion with twice the horizontal seismic intensity is 5.02mm and 8.22mm, respectively, which increases by 10.26% and 15.04% compared with that without considering vertical ground motion. Taking the bottom bending moment of pier No. 2 as an example, the vertical and transverse bending moments of pier bottom stimulated by vertical ground motion with 2 times the horizontal seismic intensity are 1373.86Kn·m and 4220.51 Kn·m, respectively, which increase by 11.96% and 9.34% compared with those without considering vertical ground motion. It can be summarized that the vertical seismic motion with twice the horizontal seismic intensity causes a maximum increase of 27.87% in the seismic response of key components, which has a slightly greater impact on displacement response than on bending moment. Therefore, the influence of vertical seismic motion cannot be ignored in the seismic response analysis of LSCGB.

### 4.3 Influence of site category

For Bridges built on different soil foundations under earthquake, on the one hand, the interaction between different soil and foundation is obviously different, resulting in the change of natural vibration period of bridges; On the other hand, due to the amplification of seismic waves, the spectral characteristics of seismic waves in bedrock are changed, which affects the dynamic response of bridges. How to evaluate the influence of site conditions on bridge structure has become a hot topic in seismic engineering. Therefore, this section selects the seismic waves corresponding to the four types of site soil specified in China’s “Urban Bridge Design Code” [27], observes the seismic response of each key component, and analyzes the influence of site types on the seismic response of LSCGB.

According to the geotechnical shear wave velocity, the site soil is divided into four categories: rock or stiff soil (V_s30_>500m/s), medium stiff soil (250< V_s30_≤500m/s), medium soft soil (140< V_s30_≤250 m/s) and soft soil (V_s30_≤140 m/s) [27]. First, pile-soil interaction under soil of different sites is determined, and the spring stiffness of each pile foundation under four soil layers obtained is shown in Table 9.

**Table 9:** Linear spring stiffness of pile group foundation in various soil sites.

Site	Position	U1	U2	U3	R1	R2	R3
category		Kn/m	Kn/m	Kn/m	Kn-m/rad	Kn-m/rad	Kn-m/rad
Stiff	Pier 1∼ 4	2.12×10^6^	2.16×10^6^	2.24×10^7^	1.56×10^8^	1.28×10^8^	1.32×10^8^
Medium stiff	Pier 1∼ 4	1.18×10^6^	1.20×10^6^	1.92×10^7^	1.33×10^8^	1.09×10^8^	1.03×10^8^
Medium soft	Pier 1∼4	6.10×10^5^	6.21×10^5^	1.05×10^7^	9.21×10^7^	7.62×10^7^	7.51×10^7^
Soft	Pier 1∼ 4	4.00×10^5^	4.10×10^5^	8.91×10^6^	6.41×10^7^	5.24×10^7^	4.26×10^7^

Note: U1, U2, and U3 represent the translational stiffness of the pile foundation; while R1, R2 and R3 represent the rotational stiffness of the pile foundation.

Finally, four bridge models considering pile-soil interaction are established, and the Ritz vector method is used for structural modal analysis to obtain longitudinal and transverse vibration periods of the whole bridge under four types of sites, as shown in Table 10. It can be seen that the natural vibration period of the LSCGB becomes longer as the soil of the site becomes softer. Compared with the first natural vibration period of the bridge on the stiff soil, the vibration period of the bridge built on the soft soil extends by 18.23% along the longitudinal direction and 16.95% along the transverse direction.

**Table 10:** Vibration period of LSCGB established in four types of soil sites (unit: s).

Vibration	Site1(Stiff)	Site2(Medium stiff)	Site3(Medium soft)	Site4(Soft)
Longitudinal-1^st^	1.141	1.185	1.215	1.349
Transverse-1^st^	0.537	0.555	0.589	0.628

**Fig 16:**
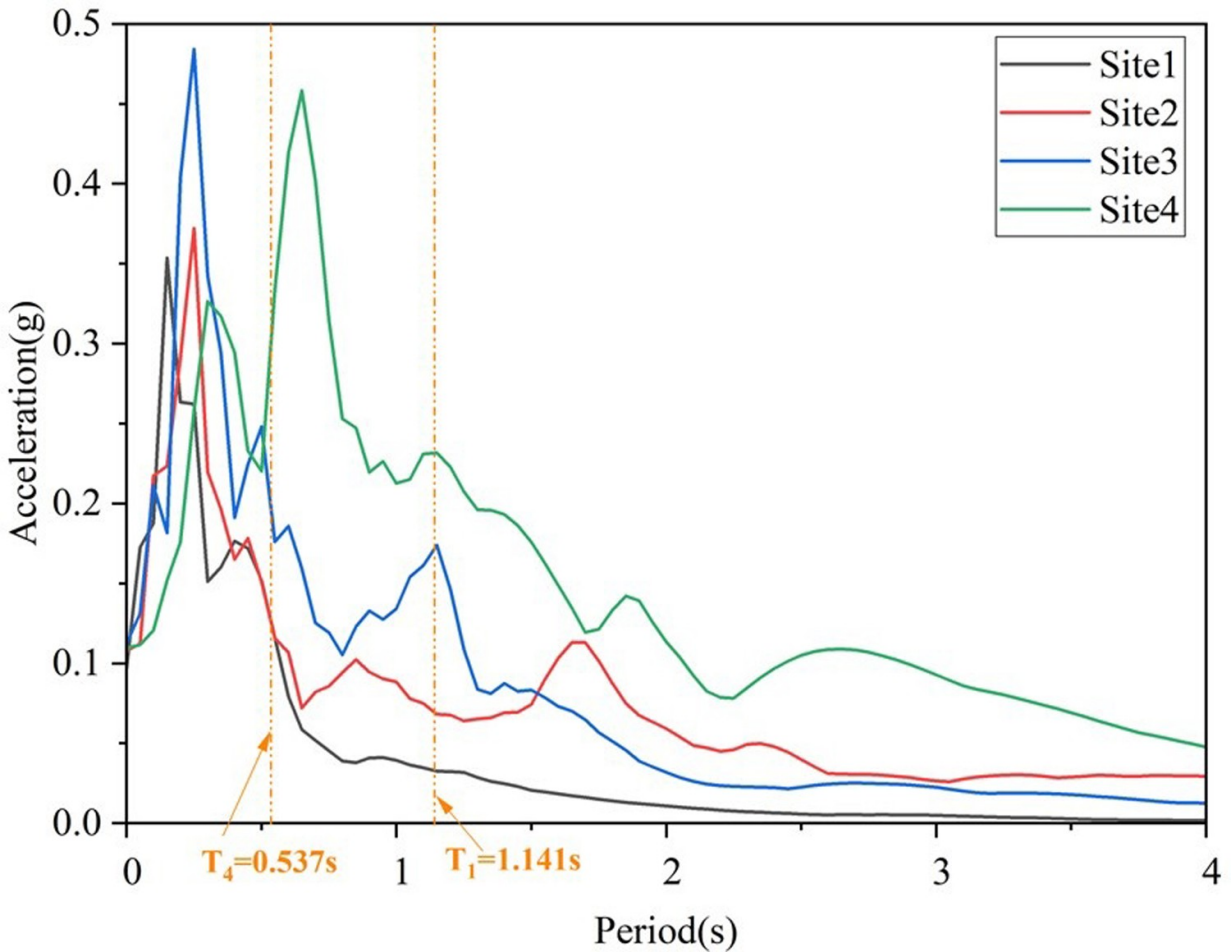
The average response spectrum of selected 7 waves of 4 site types.

According to the shear wave velocity of rock and soil, 7 seismic waves in each of four types of site soil are selected from PEER [28]. The detailed information of selected seismic waves is shown in Table 11, and the average response spectrum of 7 seismic waves in each type of site is shown in Fig 16.

**Table 11:** Detailed information of seismic waves on other sites.

Site	No.	Earthquake	Year	Station	Magnitude	Distance(km)	PGA(g)
Medium stiff	wave1	N. Palm Springs	1986	HES002	6.06	71.7	0.041
	wave2	Loma Prieta	1989	FRE000	6.93	39.32	0.127
	wave3	Landers	1992	BAK050	7.28	87.94	0.108
	wave4	Landers	1992	FTI000	7.28	62.98	0.114
	wave5	Landers	1992	PLC000	7.28	94.48	0.047
	wave6	Northridge-01	1994	MEL090	6.69	48.37	0.080
	wave7	Northridge-01	1994	OBR090	6.69	35.43	0.355
Medium soft	wave1	Borrego	1942	ELC000	6.5	56.88	0.066
	wave2	Morgan Hill	1984	G04270	6.19	11.53	0.224
	wave3	Morgan Hill	1984	HCH001	6.19	30.76	0.071
	wave4	Superstition Hills-02	1987	CAL225	6.54	27	0.190
	wave5	Loma Prieta	1989	NAS180	6.93	70.9	0.268
	wave6	Loma Prieta	1989	SFO000	6.93	58.52	0.236
	wave7	Northridge-01	1994	WBA000	6.69	66.32	0.074
Soft	wave1	Westmorland	1981	WLF225	5.9	7.57	0.195
	wave2	Westmorland	1981	WLF315	5.9	7.57	0.182
	wave3	Morgan Hill	1984	A01040	6.19	53.89	0.043
	wave4	Morgan Hill	1984	A01310	6.19	53.89	0.065
	wave5	Loma Prieta	1989	A02043	6.93	43.06	0.274
	wave6	Loma Prieta	1989	MEN270	6.93	45.42	0.110
	wave7	Northridge-01	1984	BLF206	6.69	43.22	0.176

The average peak value of the linear acceleration spectrum in the soft soil field is the largest, and the peak value of the acceleration spectrum tends to decrease with the hardening of the soil layer. The period corresponding to the peak value of the acceleration spectrum is defined as the characteristic period, and the corresponding characteristic period from the stiff soil to the soft soil is 0.15s, 0.24s, 0.26s and 0.64s, respectively, which indicates that the peak value of the acceleration spectrum and the characteristic period increase with the softening of the soil field.

**Fig 17:**
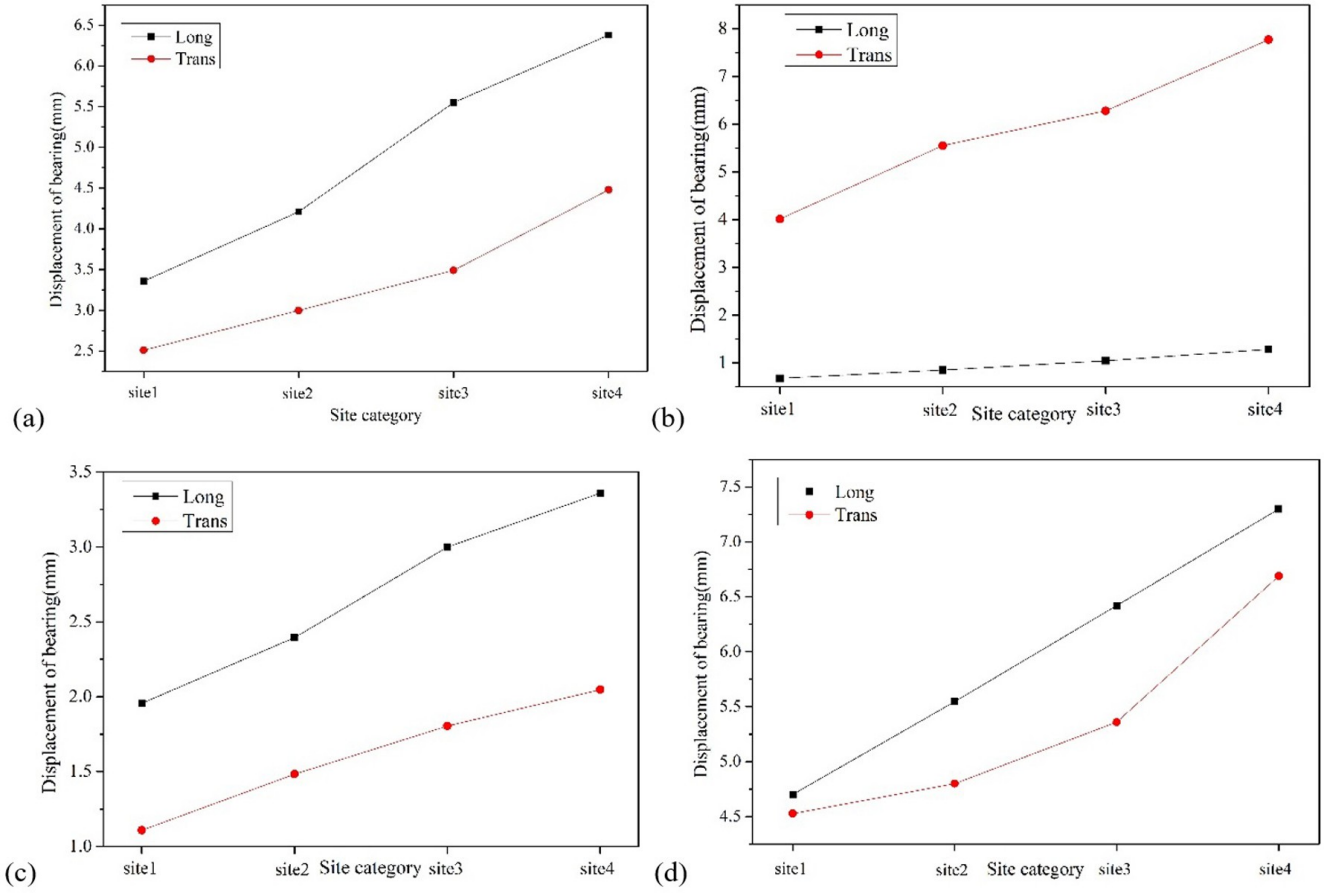
Diagram of the variation of bearing displacement with site category. (a) Bearing No. 1. (b) Bearing No.2. (c) Bearing No.3. (d) Bearing No.4.

**Fig 18:**
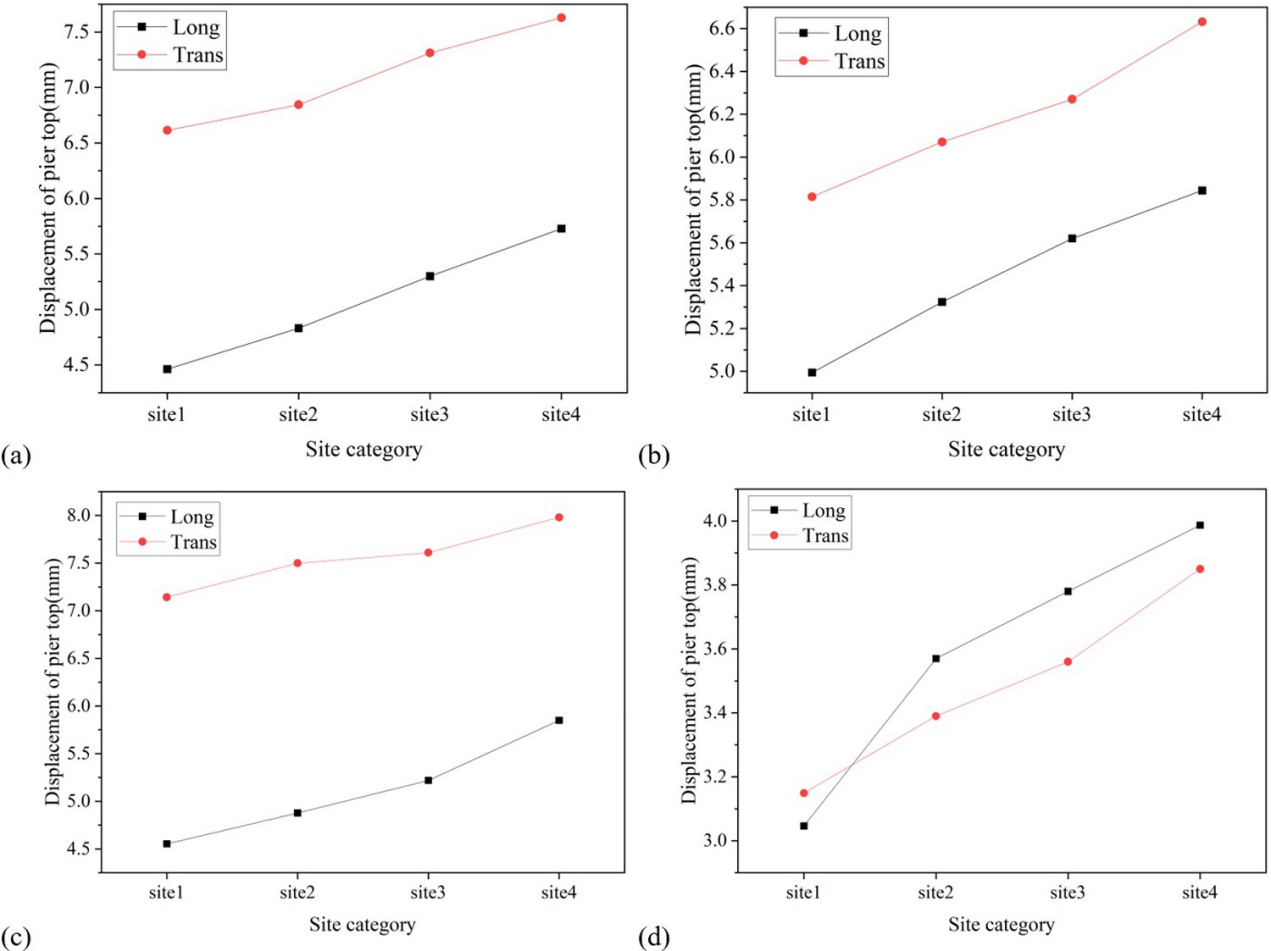
Diagram of the variation of pier top displacement with site category. (a) Pier No. 1. (b) Pier No. 2. (c) Pier No. 3. (d) Pier No. 4.

**Fig 19:**
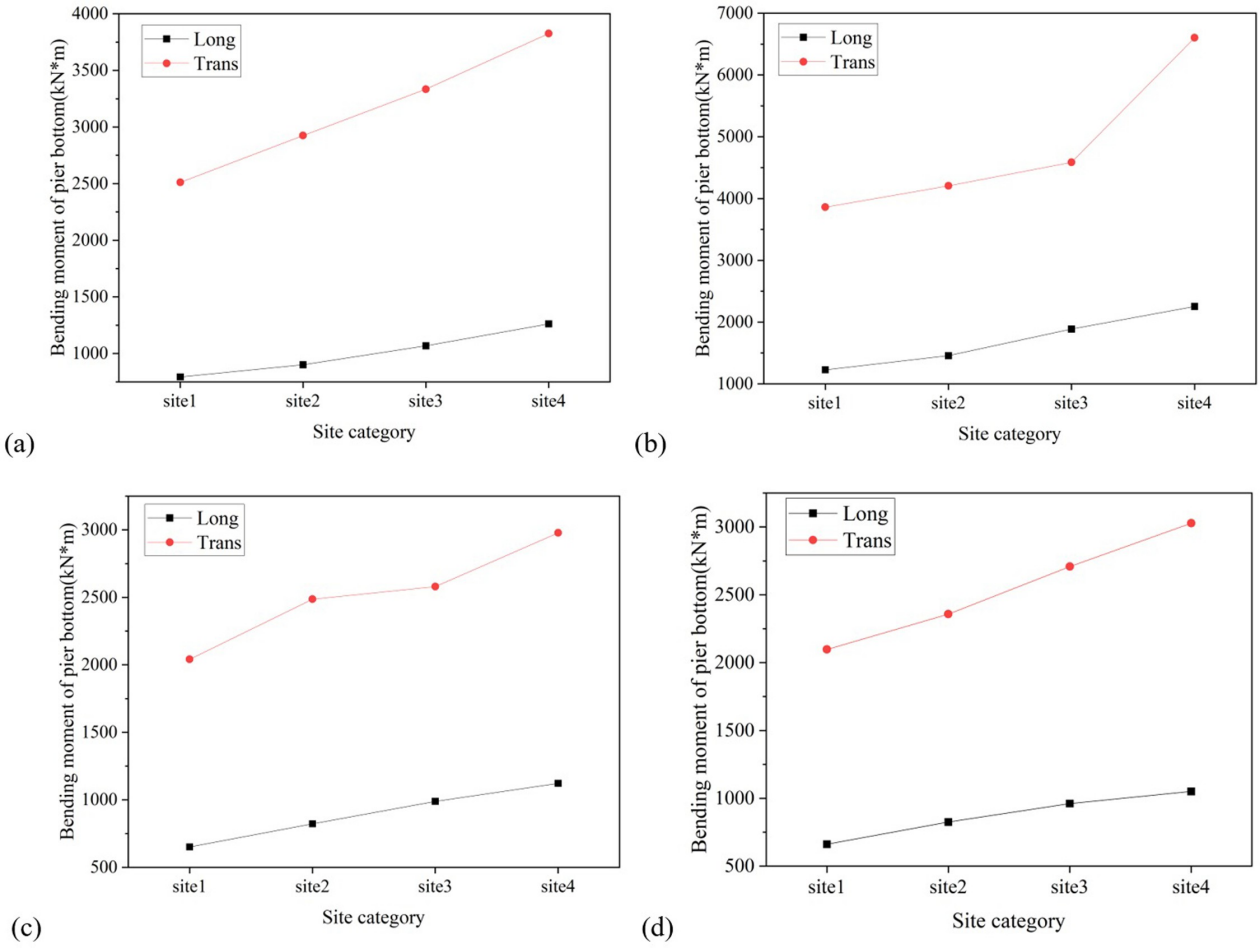
Diagram of the variation of pier bottom bending moment with site category. (a) Pier bottom No. 1. (b) Pier bottom No. 2. (c) Pier bottom No. 3. (d) Pier bottom No. 4.

The 7 seismic waves corresponding to each type of soil are respectively loaded according to the most unfavorable seismic wave incident angle (60° is selected here), and the average value of the 7 seismic waves is taken as the final response of the final engineering demand parameters. The results are listed in Figs 17–19.

Fig 17 presents that the displacement of all bearings increases with the softening of the site. Taking bearing No. 4 as an example, the longitudinal displacement of the bearing in soft soil, medium soft soil, and medium stiff soil is 7.30mm, 6.42mm, and 5.55mm, respectively, which are 1.55 times, 1.37 times, and 1.18 times the displacement of the support in stiff soil, respectively; The lateral displacement of the bearing is 6.69mm, 5.36mm, and 4.80mm, respectively, which are 1.48 times, 1.18 times, and 1.06 times the displacement of the support under stiff soil, respectively.

Similar to the displacement of the bearing, the displacement of the pier top and the bending moment at the pier bottom also increase with the softening of the site. Taking the displacement of the top of Pier No. 3 as an example, the longitudinal displacement of the top of the pier in soft soil, medium soft soil, and medium stiff soil is 5.28mm, 5.22mm, and 4.88mm, respectively, which are 1.28 times, 1.15 times, and 1.07 times the displacement of the top of the pier in stiff soil; The lateral displacement of that are 7.98mm, 7.61mm, and 7.50mm, respectively, which are 1.12 times, 1.07 times, and 1.05 times the displacement of the pier top under stiff soil, as depicted in Fig 18. Taking the bending moment at the bottom of Pier No. 2 as an example, the longitudinal bending moments at the bottom of the pier in soft soil, medium soft soil, and medium stiff soil are 2251.42 Kn·m, 1886.46 Kn·m, and 1455.46 Kn·m, respectively, which are 1.83 times, 1.54 times, and 1.19 times the bending moments at the bottom of the pier in stiff soil; The transverse bending moments of the bridge are 6602.68 Kn·m, 4586.23 Kn·m, and 4205.11 Kn·m, respectively, which are 1.71 times, 1.19 times, and 1.09 times the bending moments of the pier bottom under stiff soil, as shown in Fig 19.

The seismic response of key components increases with the softening of the site soil. This is because the soft soil site has an amplification effect on the ground motion transmitted by the bedrock, especially at the first order structural longitudinal and transverse bridge vibration periods. Compared to the soft soil site, taking the first order structural longitudinal vibration period as an example, the average seismic response spectrum value of the soft soil site during this period is 0.193g, which is 2.21 times, 2.79 times, and 7.78 times the peak acceleration spectrum of the medium soft soil, medium stiff soil, and stiff soil sites (Fig 16). On the other hand, the natural vibration period of the structure increases with the softening of the site soil, and when the foundation soil becomes soft, the natural period of the site becomes longer, so the two are more prone to resonance, ultimately amplifying the structural response.

The above analysis indicates that the site significantly affects the seismic response of the key components of the LSCGB, and the LSCGB built on the soft soil field bears greater seismic response under the action of earthquakes, that is, it is more prone to damage.

## 5 Conclusions

To obtain the influence of seismic motion parameters on the seismic response of curved girder bridges, this paper takes a steel box girder bridge with a LSCGB as an example. Firstly, a dynamic FEM is established and modal analysis is carried out. Then the influence of seismic wave incidence angle, vertical component of ground motion, and site category on its seismic response is analyzed. The following conclusions based on the case bridge are summarized:

1. The first ten vibration modes of LSCGB are mainly longitudinal, transverse and torsional vibration, and the vibration modes are dispersed. The contribution of higher-order modes to the longitudinal and transverse vibration cannot be ignored.
2. Based on the structural seismic response, it is determined that the axis formed by the central connection lines of pier No. 1 and pier No. 4 in the case bridge is the longitudinal bridge axis, and the direction perpendicular to it is the transverse reference axis. The most unfavorable horizontal seismic wave attack angle is in the direction clockwise 45°∼60° with respect to the longitudinal bridge axis.
3. The seismic response of components increases with the vertical seismic components. When the vertical seismic component reaches twice the horizontal component, the structural seismic response increases by 9.34%∼27.87% compared to not considering the vertical seismic component.
4. The seismic response of components tends to increase with the softening of the site soil. Compared to stiff site conditions, the increase in structural seismic response caused by soft soil sites ranges from 48%∼71%. Therefore, the influence of vertical component of ground motion and site category cannot be ignored when conducting seismic response analysis for LSCGBs.

On this basis, this paper can be further improved in the following two aspects, one of which is the sensitivity of seismic response of LSCGBs to the change of structural geometric parameters and the optimization of relevant structural geometric parameters, the other is to obtain the most unfavorable seismic input angle under different boundary conditions.

## Supporting information

S1 File Wave information. (XLSX)

S2 File Seismic response data of the bridge under different cases. (XLSX)
